# Biochar improved the composting quality of seaweeds and cow manure mixture and altered the microbial community

**DOI:** 10.3389/fmicb.2022.1064252

**Published:** 2022-11-24

**Authors:** Haijiang Jia, Depeng Chu, Xiangwei You, Yiqiang Li, Chongjun Huang, Jili Zhang, Xiangnan Zeng, Hui Yao, Zhaofeng Zhou

**Affiliations:** ^1^China Tobacco Guangxi Industrial Co., Ltd., Nanning, China; ^2^Marine Agriculture Research Center, Tobacco Research Institute, Chinese Academy of Agricultural Sciences, Qingdao, China

**Keywords:** biochar addition, bacterial community, metabolism function, nutrient content, organic solid wastes

## Abstract

The beneficial effects of biochar addition during composting have been proved for many feedstocks, like manures and crop straws. However, the effect of biochar on the quality of composting product with seaweed as the feedstock and the bacterial response has not been investigated. In this study, the wheat straw biochar addition on the quality of the composting product and the bacterial response was explored at the rate of 0–10%. The results showed that biochar addition at the optimal rate (5%, w/w) could increase the germination index and the ratio of the optical density of humic acid at 460 nm to that at 660 nm (E4/E6) of the composting product, which indicated the decreased biotoxicity and enhanced compost maturity. The significant increase of the nitrate nitrogen (NO_3_^−^-N) content of the composting product proved the improvement of N cycling during composting process with biochar addition. The bacterial community of composting product was shifted and the relative abundance of some beneficial taxa (e.g., *Muricauda* and *Woeseia*) was significantly increased with biochar addition. Furthermore, the relative abundance of some bacterial genes related to amino acid metabolism and carbohydrate metabolism was also increased with biochar addition. The results of our study provided the positive effect of biochar addition on the composting of seaweed and could help to produce high quality seaweed fertilizer by composting with biochar addition.

## Introduction

Because of the increasing demand of seaweeds for food consumption and chemical extraction, the production of wild and cultivated seaweed worldwide dramatically increased from 10.4 million tonnes in 2000 to 28.4 million tonnes in 2014 (Yuan et al., 2021). The increased seaweed production and utilization resulted in an increased seaweed waste, especially in the industrial process when they were deposited before processing due to the quality problem or as left over from the processing industry (Torres et al., 2019). The tremendous quantity of seaweed waste could result in serious environmental pollution and nutrients loss if not treated properly. Composting, which could produce the organic fertilizer and reduce waste volume, was treated as a sustainable and efficient management technology for utilization of the organic solid wastes (Qiu et al., 2019), including the seaweed waste. However, the peculiarities of seaweed waste as the composting feedstock, such as the relative low carbon to nitrogen (C/N) ratio, high moisture content and low porosity and potentially high salinity (Han et al., 2014) make it challenging to be composted. Therefore, the appropriate co-composted materials or amendment should be chosen and added.

Biochar, a carbonaceous material produced by pyrolyzing the organic materials under the limited oxygen condition has attracted increasing attention to be the additive during the composting (López-Cano et al., 2016). It has been evidenced that, the addition of biochar during composting could bring multiple benefits in enhancement of the composting process and the quality of compost product (Du et al., 2019a). For example, due to the high porosity structure and low density, the biochar addition could increase aeration, which result in the enhancement of microbial activities and organic matter decomposition (Guo et al., 2020) during composting. The nutrients retention in composting, especially for nitrogen (N), which was easily volatilized as ammonia could be enhanced by the increased absorption capacity with biochar addition (Malińska et al., 2014). Moreover, as a biological transformation process of organic waste, participation of microbes plays a crucial role during the composting (Qiu et al., 2019). Biochar could stimulate microbial activity and shift the microbial community by providing the habitat and altering the properties of composting mixture for microorganisms, and, finally, improve the quality of compost product (Xiao et al., 2017). The effects of biochar on the composting process and quality of compost products with different materials have been widely investigated, such as livestock manure (Mao et al., 2018), green waste (Qiu et al., 2019) and sewage sludge (Awasthi et al., 2017a; Du et al., 2019b). However, information about the effect of biochar on the composting quality of seaweed and the microbial response was limited.

In this study, we hypothesized that the addition of biochar to the mixture of seaweeds and manure could improve the quality of compost products and shift the microbial community. Therefore, the aim of this study is to (1) investigate the effect of different concentrations of biochar on the main parameters of composting products of seaweeds and manure; (2) explore the microbial response of composting products to the biochar addition.

## Materials and methods

### Description of raw composting materials

The mixture of composting materials includes seaweeds, cow manure, wheat straw and biochar. The seaweeds were provided by Qingdao Brightmoon Seaweed Group Co. Ltd. The cow manure samples were collected from a dairy from located in Jimo District, Qingdao. The wheat straw was collected from a conventional wheat farm located in Jiaozhou District, Qingdao. Furthermore, due to its enormous quantity and low value locally, wheat straw was selected as the biochar feedstock, which has been widely used in composting and recognized to contribute to the degradation of organic matter and the production of nutrient-rich compost (Zhang et al., 2016; Duan et al., 2019; Zhou et al., 2022). Biochar produced from wheat straw by pyrolyzing at 450°C for 2 h, was purchased from a biochar manufacturing Company from Shenyang, Liaoning Province, China. The basic characterization of biochar can be found in our previous study (You et al., 2022). The basic properties of the raw materials were shown in Supplementary Table S1.

### Composting process and sample collection

The composting was conducted in a rotary drum composter with the cylindrical shape. The composter was horizontally aligned and its volume was 190 l (655 mm*645 mm*480 mm). In prior to the composting process, the seaweeds were crushed into small pieces with the length less than 1 cm, the wheat straws were chopped into 0.5-cm pieces and the biochar was passed through a 0.2-cm sieve. Seaweeds and wheat straw were mixed thoroughly at the ratio of 1:1 based on dry weight, and then mixed with cow manure. In order to ensure the C/N ratio of the mixture between 25 and 30, the proportion of seaweeds: wheat straw: cow manure was mixed at 35%:35%:30% based on dry weight. Thereafter, the biochar was added into the mixture and mixed thoroughly at the ratio of 0, 2.5, 5, 10% (w/w) based on dry weight and labeled as CK, 2.5%BC, 5%BC and 10%BC, respectively. The moisture content was maintained at 60–70% by addition of water throughout the composting. There are six air vents on each longitudinal side of the composter to ensure the aeration. Additionally, the mixture was agitated once each day by rotating the rotary drum to ensure the homogenization and efficient aeration of the materials during the composting process. There are twelve composters in the experiment and each treatment was repeated for three times. After being composted for 80 days, the representative samples were collected. Each compost sample was divided into two portions. One portion was air-dried to analyze the physical and chemical characteristics, and another portion was stored at −80°C for DNA extraction.

### Physical and chemical analysis

Phytotoxicity was assayed by the Lepidium sativum test and expressed as germination index (GI), according to Zhou et al. (2019). The heavy metal concentrations were determined using atomic absorption spectrophotometer (PerkinElmer AAnalyst 800). The ratio of the optical density of humic acid at 460 nm to that at 660 nm (E4/E6) was analyzed by the spectrophotometry method. Air dried samples were used for the determination of the following parameters: electrical conductivity (EC) was measured in a 1:5 (w/v) aqueous extract using conductivity meter (DDSJ-308A, CHN). pH was determined from soil-water suspensions (1:2.5 w/v) using pH meter (AB150, Fisher Scientific, United States). The dissolved organic carbon (DOC) and dissolved organic nitrogen (DON) content in the compost were detected using a total organic carbon analyzer (multi N/C 3100, GER). The contents of elements, including total carbon (TC) and total nitrogen (TN) were determined in an Elemental analyzer (ThermoFisher FlashSmart, United States). Organic matter (OM) content was determined by potassium dichromate oxidation method. Macro-nutrients were measured after microwave HNO_3_/H_2_O_2_ digestion by Inductively Coupled Plasma spectroscopy (ICP-OES; ICAP 6500 DUO THERMO, United Kingdom). Available K was extracted with ammonium acetate and determined by flame photometer (Sherwood M410, United Kingdom). The content of nitrate nitrogen (NO_3_^−^-N) and ammonium nitrogen (NH_4_^+^-N) were extracted with 2 mol/l KCl at 1:5 (w/v), determined by segmented flow analysis (Skalar San++, France). Olsen-P was extracted with 0.5 mol/l NaHCO_3_ and determined by molybdenum blue colorimetry.

### DNA extraction and high-throughput sequencing

Genomic DNA of the final compost product was extracted using PowerSoil DNA Isolation Kit (MoBio Laboratories, Carlsbad, CA, United States) following the manufacturer’s instructions. The concentration and quality of the genomic DNA was evaluated using NanoDrop spectrophotometer (ND2000; Thermo Scientific, DE, United States) and agarose gel electrophoresis, respectively. The V3-V4 hypervariable region of 16S rRNA genes was amplified using primers 338F and 806R equipped with 12-base barcodes for sample distinction. Amplification was carried out in a 25 μl reaction solution containing 12.5 μl PCR MasterMix, 5 μM of each primer, and 5.5 μl ddH_2_O and 10 ng of template DNA. The PCR condition was set at 98°C for 1 min, followed by 30 cycles for denaturation at 98°C for 10 s, annealing at 50°C for 30 s and extension at 72°C for 30 s, with a final extension at 72°C for 5 min. The PCR product was extracted and purified using 2% agarose gels and a Qiagen DNA gel extraction kit (Qiagen, Valencia, CA), respectively. A sequencing library was generated using a TruSeq^®^ DNA PCR-Free Sample Preparation Kit (Illumina, United States). The library was applied to an Illumina NovaSeq PE250 platform by Majorbio Bio-Pharm Technology Co. Ltd. (Shanghai, China). The raw sequences were quality-filtered, and processed high-quality sequences were clustered into operational taxonomic units (OTUs) at a 97% sequence similarity level based on the UPARSE pipeline using USEARCH version 8.0 (Edgar, 2013). A representative sequence (the most abundant) of each OTU was selected for searching against the SILVA database (version 132) with a confidence cut-off value of 0.7. To eliminate the effects of different sequence numbers among the samples on the bacterial community identified, the number of sequences per sample was rarefied to the smallest sample size. The bacterial metabolic function was predicted by PICRUSt using the normalized bacterial OTU table based on the Kyoto Encyclopedia of Genes and Genomes (KEGG) database (Langille et al., 2013).

### Data analysis

All the statistical analyzes were conducted in R version 4.0.4 (R Development Core Team, 2021). One-way analysis of variance (ANOVA) was used to explore the effect of different concentrations of biochar on the physicochemical properties, bacterial OTU richness and Shannon diversity index of composting products if the data satisfy the normality of distribution and homogeneity of variance before or after log and square root transformation, then significant differences between treatments were further compared using Tukey’s honestly significant difference (HSD) test at *p* < 0.05. Otherwise, nonparametric Kruskal–Wallis test was used, followed by Conover’s test for multiple comparisons using the kwAllPairsConoverTest function in the PMCMRplus package (Pohlert and Pohlert, 2018). In order to evaluate the effect of biochar addition on the community composition of bacteria, permutational multivariate analysis of variance (PerMANOVA) was carried out using the adonis command in vegan package (Oksanen et al., 2016). Then, non-metric multidimensional scaling (NMDS) was used for the visualization of bacterial community and function composition dissimilarity among different treatments. The relative abundance of dominant functional gene families (top 40) among different treatments were depicted using the pheatmap function in the pheatmap package version 1.0.8 (Kolde, 2015). The redundancy analysis (RDA) was performed to determine the relationships between physicochemical properties and bacterial community, and the relationships between physicochemical properties and bacterial metabolic function composition of the compost using the vegan package.

## Results and discussion

### Effect of biochar on GI, E4/E6, pH and EC of the composting products

The GI values of the final composting products after 80 days were between 72.01% and 92.45% (Table 1), which indicated that the composting products were completely mature. The GI values showed an increasing trend with the increasing rate of biochar addition first and then decreased. When added with 5% biochar, the composting product has the highest GI value (92.45%), which was increased by 28.38% compared to the control. The increased GI values indicated the decreased compost biotoxicity which might be attributed to the reduced production of phytotoxic substances with biochar addition (Qiu et al., 2019). However, when biochar addition increased to a high level at 10%, the GI value decreased, which might result from the harmful compounds contained in biochar, such as polycyclic aromatic hydrocarbons, polychlorinated biphenyls and heavy metals (Odinga et al., 2021). Our results also showed that the concentration of Mg and Cd was highest in the composting product with 10% biochar addition (Table 1).

**Table 1:** Effect of biochar addition on physicochemical properties of the final composting products.

	CK	2.5%BC	5%BC	10%BC
GI (%)	72.01 ± 4.26d	81.38 ± 13.9b	91.45 ± 19.43a	75.12 ± 16.16c
Mg (mg/g)	5.12 ± 0.22b	5.51 ± 0.22b	5.45 ± 0.13b	6.41 ± 0.40a
Cd (mg/g)	0.022 ± 0.0003b	0.021 ± 0.0008b	0.022 ± 0.0002b	0.0251 ± 0.0015a
E4/E6	4.31 ± 0.04a	3.98 ± 0.25ab	3.58 ± 0.09b	3.56 ± 0.08b
pH	7.24 ± 0.03b	7.25 ± 0.02b	7.3 ± 0.04b	7.4 ± 0.01a
EC (mS/cm)	2.86 ± 0.21a	3.01 ± 0.2a	2.83 ± 0.17a	2.8 ± 0.14a
TN (%)	2.79 ± 0.29a	2.77 ± 0.07a	2.9 ± 0.07a	2.61 ± 0.14a
DON (mg/g)	0.69 ± 0.26a	1.03 ± 0.4a	0.86 ± 0.28a	0.8 ± 0.02a
NO_3_^−^-N (mg/g)	0.41 ± 0.08d	1.03 ± 0.12c	1.62 ± 0.01a	1.27 ± 0.02b
NH_4_^+^-N (mg/g)	0.29 ± 0.03a	0.35 ± 0.02a	0.32 ± 0.03a	0.3 ± 0.01a
TC (%)	26.25 ± 1.59a	28.12 ± 0.56a	30.39 ± 0.53a	30.00 ± 2.51a
OM (g/kg)	153.45 ± 0.50c	155.86 ± 0.61ab	157.24 ± 0.46a	155.06 ± 0.3b
DOC (mg/kg)	28.15 ± 1.56a	23.53 ± 1.48ab	22.6 ± 2.95ab	20.72 ± 2.26b

Data are means ± SE (*n* = 3). Different letters indicate significant difference among different treatments (*p* < 0.05). GI, germination index; E4/E6, the ratio of the optical density of humic acid at 460 nm to that at 660 nm; EC, electrical conductivity; TN, total nitrogen, DON, dissolved organic nitrogen; NO_3_^−^-N, nitrate nitrogen; NH_4_^+^-N, ammonium nitrogen; TC, total carbon; OM, organic matter; DOC, dissolved organic carbon. CK, compost without biochar; 2.5%BC, compost added with 2.5% (w/w) biochar; 5%BC, compost added with 5% (w/w) biochar; 10BC%, compost added with 10% (w/w) biochar.

The E4/E6 ratio, which was measured by the optical density of a humic acid solution at 465 and 665 nm inversely reflect the degree of compost humification. A low E4/E6 value indicates the high degree of mature and stabilization, and a low content of aliphatic structures. In this study, the E4/E6 values of composting products decreased by 7.55%–17.35% with the increasing rate of biochar addition to 10% (w/w; Table 1), which indicated that the biochar addition could enhance the compost maturity. The stimulation of microbial abundance and biomass by altering the composting properties and providing habitat with biochar addition might attribute to the enhanced condensation of aromatic humic constituents during composting, which was beneficial to the improvement of compost quality.

The pH values usually increased during the initial stage of composting due to the consumption of organic acids. Thereafter, with the decomposition of organic material which produce organic acids, the pH values decreased gradually (Zhou et al., 2019). In this study, the pH values of the final composting products were 7.24–7.40 (Table 1). The effect of biochar addition at low rates on the pH was not significant. However, when added at 10%, the pH of the composting products was significantly increased due to the alkalinity of biochar.

EC, which reflects the content of soluble salts is usually used to indicate the potential phytotoxicity of the composting products. Despite properties of the raw materials, the EC values were usually related to the releasing and precipitation of minerals, water evaporation and ammonia volatilization during the composting process (Zhang et al., 2016). The results of this study showed that, biochar addition had no significant effect on EC values of the composting products, which were in the range of 2.80–3.01 mS/cm (Table 1). During the entire composting process, the safety threshold of 4 mS cm^−1^ was never exceeded.

### Effect of biochar on nutrients and minerals of the composting products

#### Changes in TN, DON, NO_3_^−^-N and NH_4_^+^-N

The responses of TN, DON, NO_3_^−^-N and NH_4_^+^-N contents of final compost product to biochar addition are shown in Table 1. In this study, the content of TN of the final compost products were 2.2–3.2% (Table 1), which is similar with the TN content of the biochar amended dewatered fresh sewage sludge-wheat straw co-composting (Awasthi et al., 2017b). Some previous studies have shown that the TN contents of final compost product increased significantly after biochar addition (Zhou et al., 2022). However, biochar addition had no significant effect on the TN content in current study. The response of final compost TN to biochar addition was significantly influenced by composting conditions, such as compost method, initial carbon to nitrogen ration and initial moisture content (Zhou et al., 2022), which may vary dramatically among different material and studies.

As an important fraction of dissolved organic matter, the average concentrations of DON were 0.68 mg/g, 1.03 mg/g, 0.86 mg/g and 0.80 mg/g with 0, 2.5, 5 and 10% biochar added, which were slightly higher with biochar addition than control, but not significantly (Table 1). Similarly, results of previous studies showed that compost mixed with biochar was a feasible approach to increase important nutrients, such as DON and NO_3_^−^ (Awasthi et al., 2017b; Nguyen et al., 2022).

As shown in Table 1, biochar addition significantly increased the content of NO_3_^−^-N, indicating the improvement of N cycling with biochar addition, which was in accordance with many previous studies (López-Cano et al., 2016; Wang et al., 2021; Nguyen et al., 2022; Zhou et al., 2022). The final composting product added with 5% biochar had the highest NO_3_^−^-N content, which was increased by about three times compared to the control. This phenomenon is probably because that biochar addition could improve the pore structure and aeration of compost substrate, owing to their characteristics, such as high porosity, low density and large specific surface area, thus subsequently promote the growth and reproduction of nitrifying microorganisms (Godlewska et al., 2017). However, biochar addition had no significant effect on the NH_4_^+^-N content, which ranged 0.24–0.37 mg/g (Table 1). The influence of biochar addition on the NH_4_^+^-N and NO_3_^−^-N contents is of great importance for the quality and application potential of compost product. The nitrification index, that is the ratio of NH_4_^+^-N to NO_3_^−^-N, can also be used as an indicator of compost maturity (Cáceres et al., 2018). The decrease in nitrification index of the final compost product with biochar addition indicates a positive improvement in compost maturity. Furthermore, as NO_3_^−^-N is a better form of N for many plants than NH_4_^+^-N, a high nitrate concentration in compost is normally desired to improve the compost quality (Sun et al., 2016; Cáceres et al., 2018).

#### Changes in TC, OM and DOC

The effects of biochar addition on the contents of TC, OM and DOC are shown in Table 1. Biochar addition slightly increased the TC content, from 26% in control to 30% with 5% biochar addition (Table 1). As shown in Table 1, the OM content was significantly increased by biochar addition. When added with 5% biochar, the composting product has the highest OM content (157.24 g/kg). This increase of OM after biochar addition can be explained by two reasons. Firstly, biochar is a carbon-rich material and contains an amount of organic matter. Thus, the direct input of organic matter that biochar itself contained may increase the OM content of the compost. Second, biochar addition can accelerate the formation of humus during the composting, which may also increase the OM content of the compost (Liu et al., 2021; Wang et al., 2022).

The DOC is the most direct carbon source that can be decomposed and utilized by microorganisms in the compost mass, and is one of the most important indicators of microbial activities and rate of composting. The DOC content was decreased by biochar addition from 5.63 mg/kg in control to 4.14 mg/kg in treatment amended with 10% biochar (*p* < 0.05; Table 1). A previous study also reported a decrease of DOC content after wheat straw biochar added to pig manure (Zhang et al., 2016). Due to its high porosity, biochar addition could decrease the composting density, enhance the aerobic condition, as well as provide comfortable shelters for microorganisms and improve microbial activity, thus promoting DOC decomposition (Khan et al., 2014), which could account for the decrease in DOC concentration in the biochar addition treatments. Besides, the porous structure and large surface area of biochar endowed them with high sorption, which could absorb the DOC, resulting the reduction in DOC content. Notably, the DOC concentration in this study met the requirement (<10 g/kg) for stability and maturity reported by previous study (Wang et al., 2017).

#### Changes in TK, TP, available K and Olsen-P

The changes of TK, TP, available K and Olsen-P concentrations to biochar addition were shown in Supplementary Figure S1. The content of TK, TP, available K and Olsen-P in different treatments ranged from 1.75 ± 0.09% to 1.82 ± 0.02%, from 0.25 ± 0.03% to 0.35 ± 0.04%, from 12.15 ± 0.18 to 12.83 ± 0.08 g/kg, and from 3,126 ± 622.8 to 3,609 ± 179.9 mg/kg, respectively, which were all not significantly affected by biochar addition. These elements cannot be transformed into gaseous material, and are not easily to lose during the composting, thus are not susceptible to biochar addition.

### Effect of biochar on bacterial communities of the composting products

**Figure 1:**
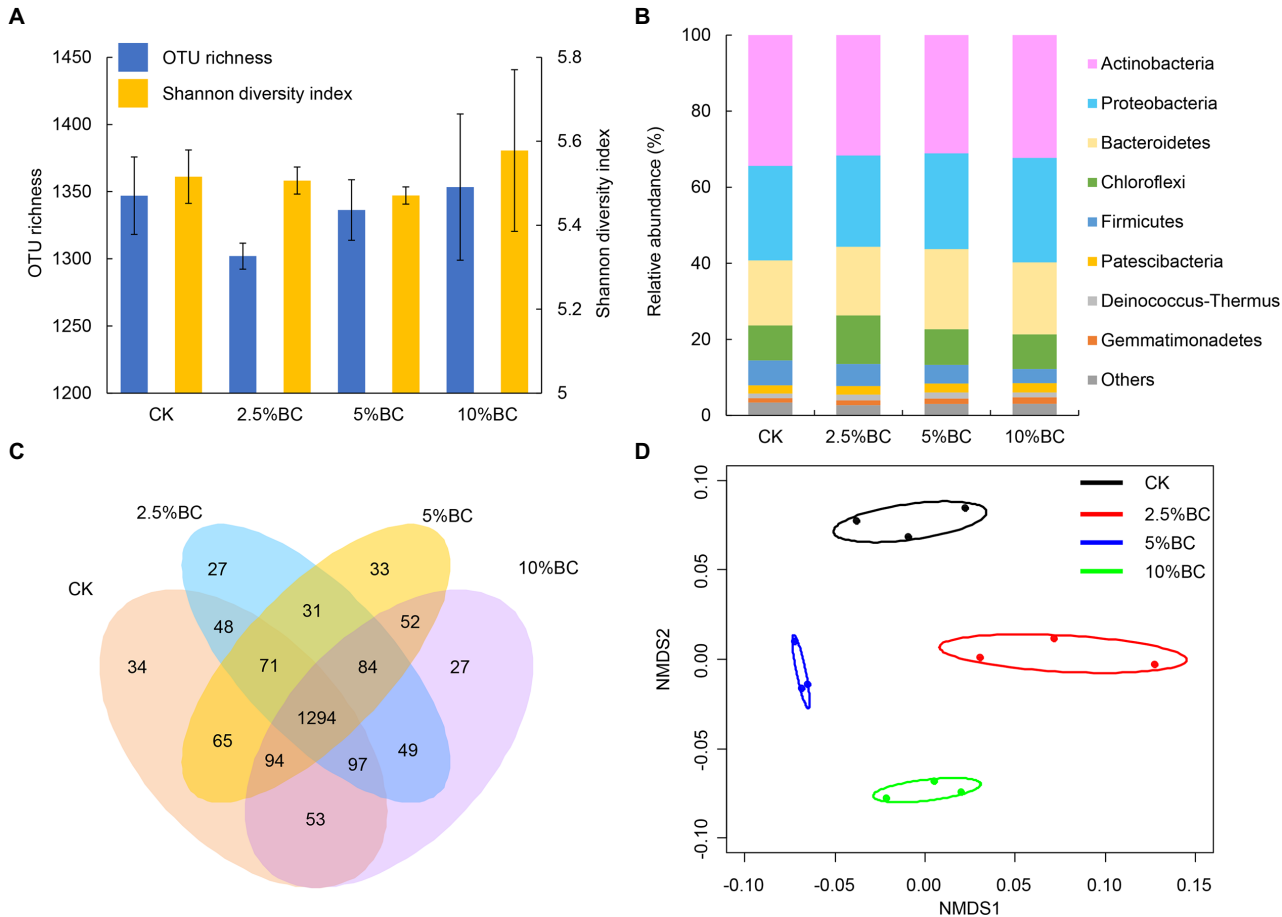
Effect of biochar on the bacterial community profiles of the compost. **(A)** Operational taxonomic unit (OTU) richness and Shannon diversity index. **(B)** Relative abundance of bacteria at phylum level. **(C)** Venn diagram showing the number of specific and shared OTUs in different treatments. **(D)** Nonmetric multidimensional scaling (NMDS) ordination plots of bacterial community composition in different treatments based on the Bray-Curtis distance similarity. The bacterial phyla represent <0.5% of the total bacterial sequences and are not identified to phylum level were all assigned to “Others.” Ellipses in the plots denote 95% confidence intervals for the centroids of different treatments. CK, compost without biochar; 2.5%BC, compost added with 2.5% (w/w) biochar; 5%BC, compost added with 5% (w/w) biochar; 10%BC, compost added with 10% (w/w) biochar.

The bacterial OTU richness and Shannon diversity index of the final composting products after 80 days in different treatments ranged from 1,302 ± 9.6 to 1,353 ± 54.4 and 5.47 ± 0.02 to 5.58 ± 0.19, respectively (Figure 1A). One-way ANOVA showed that biochar addition had no significant effect on the bacterial OTU richness (*F* = 1.431, *p* = 0.304) and Shannon diversity index (χ^2^ = 1.564, *p* = 0.668). The effect of biochar addition on bacterial diversity of compost were inconsistent in different studies. For example, a positive effect of biochar addition on bacterial diversity has been observed during rice straw composting with pig manure (Zhou et al., 2019), pig manure and wheat straw composting (Awasthi et al., 2020), and chicken manure mixed with peanut straw composting (Li et al., 2022). However, Awasthi et al. (2017a) found a negative effect of biochar addition on the bacterial diversity during sewage sludge composting at the thermophilic phage. This inconsistent effect of biochar addition on microbial diversity may be caused by the differences in the properties of the compost raw materials, feedstock type and addition rate of biochar.

The relative abundances of bacterial taxa at the phylum level are shown in Figure 1B. The dominant taxa were Actinobacteria and Proteobacteria, followed by Bacteroidetes, Chloroflexi and Firmicutes, which together accounting for more than 90% of the total bacterial sequences (Figure 1B). Previous studies also reported that some of these phyla were the most predominant in biochar amended compost with different raw materials and biochar dosages (Yang et al., 2020; Li et al., 2022) and play important roles during the composting. For example, Proteobacteria played a vital role in the degradation of compost and was related to the nitrogen and carbon cycling (Awasthi et al., 2017a; Zhou et al., 2019). Actinobacteria can contribute to the degradation of persistent compounds, such as lignocelluloses (Taha et al., 2016) and promote the elimination of pathogenic microbes by secreting many antibiotics (Franke-Whittle et al., 2009). Firmicutes can secrete many kinds of extracellular enzymes, playing important role in the degradation of protein and cellulose (Zhu et al., 2021).

**Figure 2:**
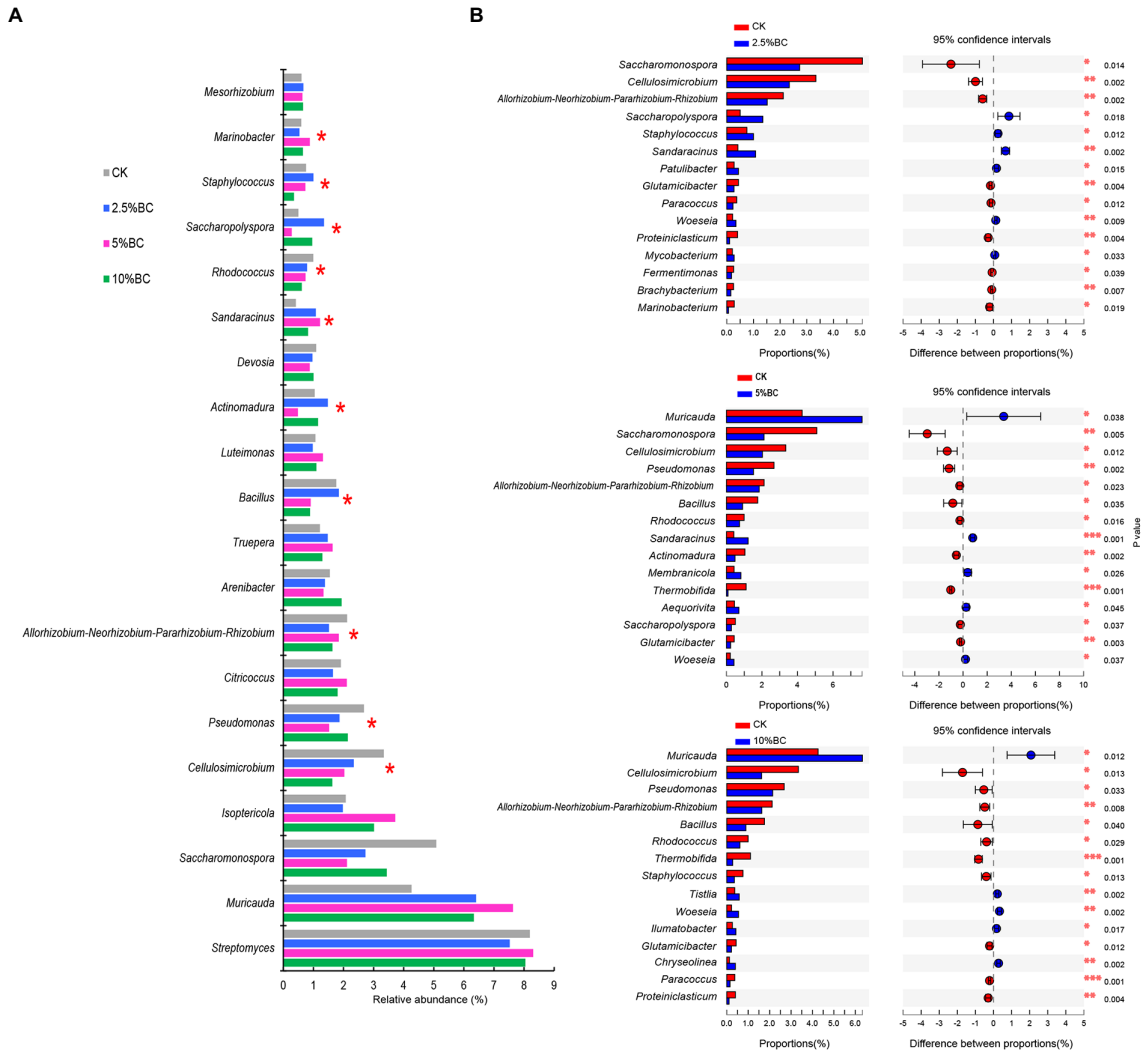
Effect of biochar on the bacterial relative abundance at genus level. **(A)** The relative abundance of top 20 dominant bacterial genera in different treatments. **(B)** Two group comparison based on genus level between CK and biochar addition treatment. CK, compost without biochar; 2.5%BC, compost added with 2.5% (w/w) biochar; 5%BC, compost added with 5% (w/w) biochar; 10%BC, compost added with 10% (w/w) biochar.

Venn diagram showed that there were 34, 27, 33 and 27 unique OTUs observed in CK, 2.5%BC, 5%BC and 10%BC treatment, respectively (Figure 1C), indicating that the bacterial community compositions of the final compost products were different among different treatments, which was confirmed by the NMDS ordination (Figure 1D). Similarly, PerMANOVA showed that biochar addition significantly affected the bacterial community composition of the compost (*F* = 2.418, *R*^2^ = 0.476, *p* = 0.001). Furthermore, the relative abundances of bacterial genera (top 20) in different treatments are shown in Figure 2A, in which ten genera, including *Cellulosimicrobium*, *Pseudomonas*, *Allorhizobium*-*Neorhizobium*-*Pararhizobium*-*Rhizobium*, *Bacillus*, *Actinomadura*, *Sandaracinus*, *Rhodococcus*, *Saccharopolyspora*, *Staphylococcus*, and *Marinobacter* showed significant difference in the relative abundance among different treatments, verifying the significant difference of bacterial community composition among different treatments. Previous studies also reported that biochar addition significantly influenced microbial community structure of the rice straw-pig manure (Zhou et al., 2019), cattle manure-maize straw composting (Bello et al., 2020) and chicken manure mixed with peanut straw composting (Li et al., 2022). The variation in the bacterial community composition among different treatments may be ascribed to different concentrations of biochar added would regulate the composting microhabitats, change the physicochemical properties of compost, thereby, influence the microbial activity and community composition.

To further investigate the response of bacterial taxa to biochar addition, the top 15 bacterial genera showing significant difference in the relative abundance of compost with or without biochar addition are shown in Figure 2B. Compare with CK, there were 6 (*Saccharopolyspora*, *Staphylococcus*, *Sandaracinus*, *Patulibacter*, *Woeseia*, and *Mycobacterium*), 5 (*Muricauda*, *Sandaracinus*, *Membranicola*, *Aequorivita* and *Woeseia*), and 5 genera (*Muricauda*, *Tistlia*, *Woeseia*, *Ilumatobacter*, and *Chryseolinea*) with a significantly higher relative abundance in the 2.5%BC, 5%BC and 10%BC treatment, respectively, indicating that biochar addition promoted the growth of these taxa. Interestingly, some genera, such as *Muricauda* and *Woeseia*, often isolated from ocean or sea intertidal sediment, were enriched after biochar addition in current study, probably because present study used seaweeds as the feedstocks, which is different from previous studies. Members of these genera have been reported to have important ecosystem functioning. For example, the strain of the *Muricauda* was reported to exhibit strong quorum quenching activity, making it a potential biocontrol agent (Tang et al., 2013). *Woeseia* could carry out diverse ecological functions like dissimilatory sulfur oxidation and denitrification (Mußmann et al., 2017), which could contribute a lot to biogeochemical cycling of nutrients. However, their roles in seaweeds composting are poorly documented and should be further investigated.

### Effect of biochar on microbial metabolism functions of the composting products

**Figure 3:**
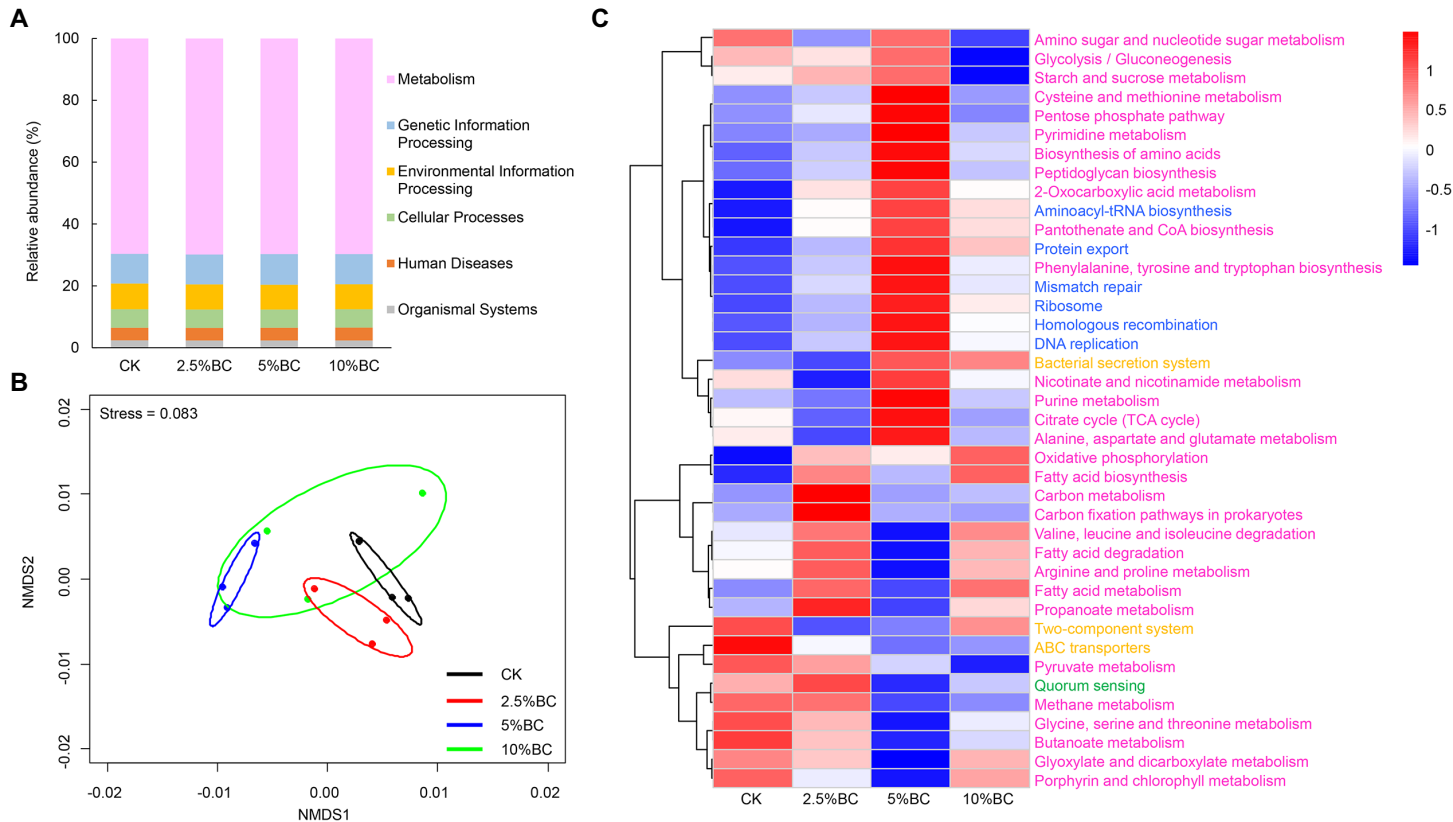
Effect of biochar on bacterial metabolic function profiles of the compost analyzed by PICRUSt. **(A)** Relative abundance of six function categories. **(B)** NMDS ordination plots of bacterial function composition in different treatments based on the Bray-Curtis distance similarity. **(C)** Heat map showing the relative abundance of the top 40 functional gene families predicted by PICRUSt. Gene families are colored by functional categories. CK, compost without biochar; 2.5%BC, compost added with 2.5% (w/w) biochar; 5%BC, compost added with 5% (w/w) biochar; 10%BC, compost added with 10% (w/w) biochar.

To explore the effect of biochar addition on bacterial function of the final compost products, we used PICRUSt to perform bacterial function prediction analysis based on the Kyoto Encyclopedia of Genes and Genomes (KEGG) database. Six categories of biological metabolic pathways, including metabolism, genetic information processing, environmental information processing, cellular processes, organ systems, and human diseases were obtained. Among these pathways, metabolism was the most primary, whose relative abundance was about 70%, followed by genetic information processing (9.7%), environmental information processing (8%), and cellular processes (5.9%; Figure 3A). NMDS ordination showed that the bacterial function compositions in different treatments were separate with each other (Figure 3B), indicating that biochar addition at different concentrations significantly affect the bacterial metabolism functions of the final compost products. Furthermore, the heatmap showed that compared with CK, biochar addition, especially at 5% rate, increased the relative abundance of some bacterial genes associated with amino acid metabolism (e.g., cysteine and methionine metabolism, alanine, aspartate and glutamate metabolism, and phenylalanine, tyrosine and tryptophan biosynthesis), and carbohydrate metabolism (e.g., glycolysis/gluconeogenesis, amino sugar and nucleotide sugar metabolism, citrate cycle (TCA cycle), pentose phosphate pathway, and starch and sucrose metabolism; Figure 3C). Previous studies also illustrated that biochar addition significantly increased the abundance of sequences related to amino acid metabolism and carbohydrate mentalism (Zhou et al., 2019). By regulating the carbohydrate mentalism, biochar addition can influence the production of various compounds that is associated with the decomposition of cellulose and hemicellulose (Toledo et al., 2017). Furthermore, during the composting process, amino acids can be produced, which is also the energy and carbon source of bacterial diversity and metabolism. Therefore, the increase relative abundance of bacteria related with amino acid metabolism induced by biochar addition may contribute to the acceleration on amino acids production and humic substance synthesis (Wu et al., 2017).

### Relationship between physicochemical properties, bacterial communities and metabolic functions

**Figure 4:**
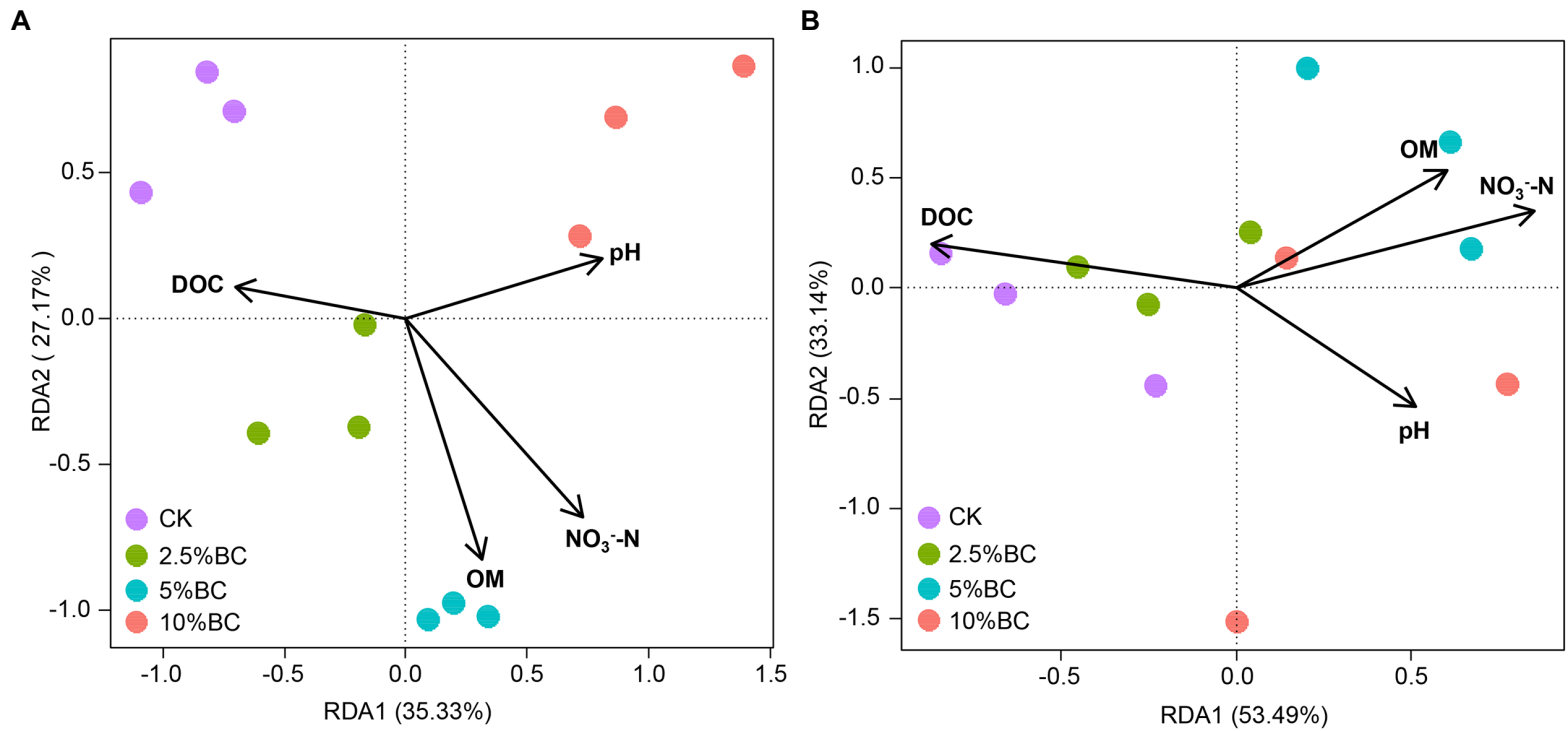
Redundancy analysis (RDA) revealing the relationship of selected physicochemical properties with **(A)** the bacterial community and with the **(B)** metabolic function of bacterial communities. CK, compost without biochar; 2.5%BC, compost added with 2.5% (w/w) biochar; 5%BC, compost added with 5% (w/w) biochar; 10 BC%, compost added with 10% (w/w) biochar.

The physicochemical properties could influence bacterial communities and metabolism during composting. Four main properties (pH, OM, DOC and NO_3_^−^-N) changed after biochar addition were selected to investigate the relationship of physicochemical properties of the compost with the bacterial community and the metabolic functions using RDA (Figure 4). The results showed that the physicochemical properties selected in this study largely explained the variations of the bacterial community composition (62.5% of the total variation explained by RDA1 and RDA2) and metabolic function composition (86.6%). NO_3_^−^-N (*R*^2^_community_ = 0.98, *p* = 0.001; *R*^2^_metabolic_ = 0.66, *p* = 0.011), OM (*R*^2^ = 0.79, *p* = 0.001; *R*^2^ = 0.44, *p* = 0.086), pH (*R*^2^ = 0.72, *p* = 0.007; *R*^2^ = 0.44, *p* = 0.077) and DOC (*R*^2^ = 0.45, *p* = 0.063; *R*^2^ = 0.71, *p* = 0.006) were significantly or marginally significantly influence the bacterial community and metabolic function composition of the final compost products, which were also reported in rice straw composting with pig manure amended with biochar (Zhou et al., 2019), pig manure composing amended with bean dregs and biochar (Yang et al., 2020), and in paper mill sludge composting amended with animal-derived and plant-derived biochar (Liu et al., 2022). Certain microbial taxa can decompose organic matter and acquire N from the composting and reproduce with appropriate C:N stoichiometric ratios (Li et al., 2017). Therefore, the quality and quantity of the nutrients, such as C and N source will surely influence the bacterial communities and its metabolic functions. These results revealed that compost physicochemical properties shifted by biochar addition will influence the bacterial community and metabolic function, which may regulate the organic matter transformation during composting.

## Conclusion

In this study, the effect of biochar amendments on the quality of the final composting products with seaweed as the feedstock and the responses of bacterial community and metabolic functions were investigated. Biochar addition significantly decreased biotoxicity, enhanced maturity, and influenced some physicochemical properties of the final compost product. Furthermore, biochar addition significantly affected the bacterial community composition, especially by increasing the relative abundance of some beneficial taxa. Some genes related with amino acid and carbohydrate metabolism were also enhanced by biochar addition. Additionally, NO_3_^−^-N content is an important factor influencing the bacterial community and metabolic composition. The findings of our study provided the positive effect of biochar addition on the composting of seaweed and could help to produce high quality seaweed fertilizer by composting with biochar addition. In addition, we should realize that this study only investigates the effect of biochar addition on the quality and microbial community of the final composting products, dynamic investigation should be conducted in the further study, which is helpful to understand the change process of the whole experiment.

## Data availability statement

The datasets presented in this study can be found in online repositories. The data presented in the study are deposited in the Genome Sequence Archive (GSA) repository, accession number CRA008555.

## Author contributions

XY, YL, ZZ, and HJ conceived and designed the experiments. DC, XY, CH, JZ, and XZ conducted the experiments. HY conducted the data analysis and wrote the first manuscript with DC and HJ. ZZ and HJ reviewed and edit the manuscript. All authors contributed to the article and approved the submitted version.

## Funding

This work was financially funded by the project of the China Tobacco Guangxi Industrial Co., Ltd., “Research and application of biochar seaweed organic fertilizer for controlling tobacco root diseases” (contract number 2020450000340002).

## Conflict of interest

HJ, CH, JZ, XZ, and ZZ are employed by China Tobacco Guangxi Industrial Co., Ltd.

This study received funding from China Tobacco Guangxi Industrial Co., Ltd. The funder had the following involvement in the study: study design, data collection, and the decision to submit for publication. The remaining authors declare that the research was conducted in the absence of any commercial or financial relationships that could be construed as a potential conflict of interest.

## Publisher’s note

All claims expressed in this article are solely those of the authors and do not necessarily represent those of their affiliated organizations, or those of the publisher, the editors and the reviewers. Any product that may be evaluated in this article, or claim that may be made by its manufacturer, is not guaranteed or endorsed by the publisher.
