# Supplementary material for: Biochar improved the composting quality of seaweeds and cow manure mixture and altered the microbial community
Source: Front Microbiol. 2022 Nov 24;13:1064252. doi: 10.3389/fmicb.2022.1064252 (PMC9731296; doi:10.3389/fmicb.2022.1064252)
Supplement: Supplementary file 1 [file Data_Sheet_1.docx]

**Supplementary Information for**

**Biochar improved the composting quality of seaweeds and cow manure mixture and altered the microbial community**

**Haijiang Jia^1†^, Depeng Chu^2†^, Xiangwei You^2^, Yiqiang Li^2^, Chongjun Huang^1^, Jili Zhang****^1^, Xiangnan Zeng^1^, Hui Yao^2*^, Zhaofeng Zhou^1*^**

^1^China Tobacco Guangxi Industrial Co., Ltd., Nanning 530001

^2^ Marine Agriculture Research Center, Tobacco Research Institute, Chinese Academy of Agricultural Sciences, Qingdao 266101, China

†These authors contributed equally to this work and share first authorship

**Supplementary Table 1**

**Supplementary Figure 1**

**Supplementary Tables**

| **Supplementary Table 1.** Basic properties of the composting materials used in this study. | | | | |
| --- | --- | --- | --- | --- |
| Parameters | Seaweeds | Wheat straw | Cow manure | Biochar |
| Moisture content (%) | 22.42 | 8.16 | 41.76 | 14.04 |
| pH | 6.86 | 5.12 | 7.14 | 9.75 |
| Electrical conductivity (mS/cm) | 4.70 | 6.21 | 2.42 | 2.46 |
| C:N | 12.18 | 26.13 | 20.12 | 43.45 |
| NO_3_^-^-N (mg/g) | 0.03 | 0.94 | 1.90 | 0.06 |
| NH_4_^+^-N (mg/g) | 0.61 | 0.25 | 0.15 | 0.03 |

**Supplementary Figures**


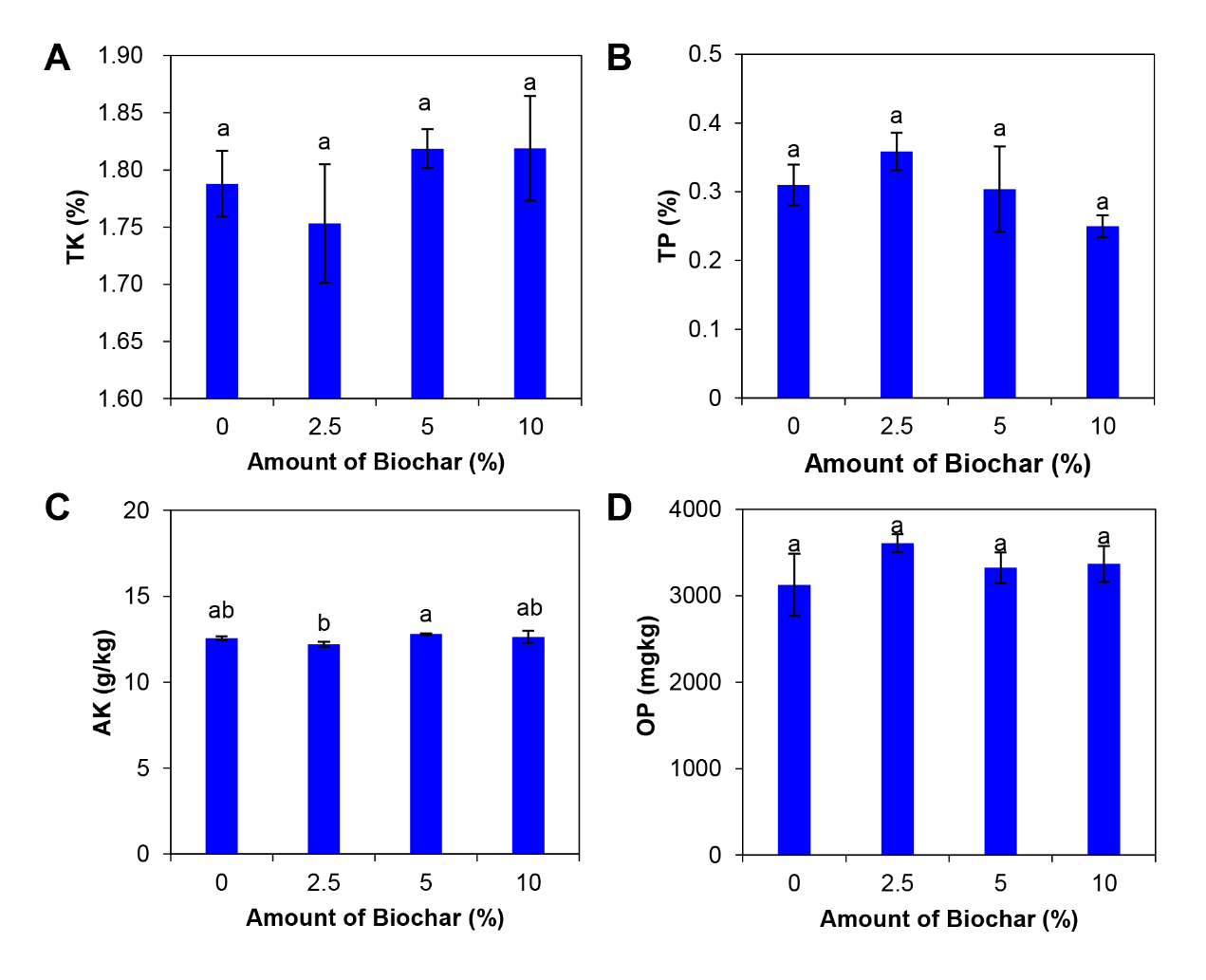


**Supplementary Figure 1.** Effect of biochar on (A) Total potassium (TK), (B) total phosphorus (TP), (C) available potassium (AK) and (D) Olsen-P (OP) of the compost. Data are means ± SE (n = 3). Different letters indicate significant difference among different treatments (*P* < 0.05).
